# Supplementary material for: MisTIC: Missegmented Transcript Inference Correction for Improved Spatial Transcriptomics Analysis
Source: bioRxiv. 2025 Dec 15:2025.12.11.693759. Preprint. [Version 1] doi: 10.64898/2025.12.11.693759 (PMC12724677; doi:10.64898/2025.12.11.693759)
Supplement: Supplement 2 [file media-2.pdf]

Platform	Disease	Type	Data link
Xenium	LUAD	SRT	https://www.10xgenomics.com/datasets/ffpe-human-lung-cancer-data-with-human-immuno-oncology-profiling-panel-and-custom-add-on-1-standard
MERSCOPE	LUAD	SRT	https://console.cloud.google.com/storage/browser/vz-ffpe-showcase/HumanLungCancerPatient1;tab=objects?pageState=(%22StorageObjectListTable%22:(%22f%22:%22%255B%255D%22))&prefix=&forceOnObjectsSortingFiltering=false
MERSCOPE	HCC	SRT	https://console.cloud.google.com/storage/browser/vz-ffpe-showcase/HumanLungCancerPatient1;tab=objects?pageState=(%22StorageObjectListTable%22:(%22f%22:%22%255B%255D%22))&prefix=&forceOnObjectsSortingFiltering=false
MERSCOPE	Prostate Cancer	SRT	https://console.cloud.google.com/storage/browser/vz-ffpe-showcase/HumanProstateCancerPatient1;tab=objects?pageState=(%22StorageObjectListTable%22:(%22f%22:%22%255B%255D%22))&prefix=&forceOnObjectsSortingFiltering=false
CosMX	Pancreas	SRT	https://nanostring.com/products/cosmx-spatial-molecular-imager/ffpe-dataset/cosmx-smi-human-pancreas-ffpe-dataset/
Xenium Prime	Melanoma	SRT	https://www.10xgenomics.com/datasets/xenium-prime-ffpe-human-skin
Xenium Prime	Prostate Cancer	SRT	https://www.10xgenomics.com/datasets/xenium-prime-ffpe-human-prostate
TCGA	LUAD	Bulk RNA-seq	https://www.cancer.gov/ccg/research/genome-sequencing/tcga
scRNA-seq	LUAD	scRNA-seq	https://www.ncbi.nlm.nih.gov/geo/query/acc.cgi?acc=GSE97168
