# Supplementary material for: MisTIC: Missegmented Transcript Inference Correction for Improved Spatial Transcriptomics Analysis
Source: bioRxiv. 2025 Dec 15:2025.12.11.693759. Preprint. [Version 1] doi: 10.64898/2025.12.11.693759 (PMC12724677; doi:10.64898/2025.12.11.693759)

[illegible]

Fig. S1 Cell type distribution in the Xenium and MERSCOPE LUAD scSRT datasets. (a) Xenium LUAD, (b) MERSCOPE LUAD. Cells are plotted based on their centroid coordinates, with colors indicating cell types.

Fig. S2 Scatter heatmaps showing mutually exclusive expression patterns for selected gene pairs. (a) ACTA2 (fibroblasts) vs CD3E (T cells); (b) EPCAM (epithelial cells) vs CD68 (macrophages); (c) CD3E (T cells) vs MRC1 (macrophages). Only cells expressing at least one gene in each pair are included. Each box represents an expression bin defined by the respective gene expression levels, with color intensity mapped logarithmically to the fraction of cells in that bin.

Fig. S3. Benchmarking Traditional Doublet Detection Algorithms in Simulated Datasets. (a) False positive rate (FPR)-true positive rate (TPR) plots for DoubletDetection (left) and Scrublet (right), colored by dataset. Inset plots on 1-to-1 x- and y-axis scale, positioned so as to not cover any data points on the larger graph. (b) Results from tests using default values for DoubletDetection (louvain algorithm, 0.5 voter threshold) and the top five best performing results (highest F_1 score) for each of the six datasets are shown in the left side plots colored in red. Results from tests using default values for Scrublet (0.06 expected doublet rate) and top five best performing results are shown in the right side plots colored in red. (c) Bar graphs showing mean TPR for DoubletDetection when using differing datasets and parameters. From left to right: dataset, algorithm selection, and voter threshold. (d) Bar graphs showing mean TPR for Scrublet. From left to right: dataset, threshold, and expected doublet rate.

Fig. S4 Scatter heatmap showing genes expressed exclusively in cells. Only cells expressing at least one gene in each pair are included. Each box represents an expression bin defined by the expression level of one gene in the pair, with bin colors scaled logarithmically to the fraction of cells.

Fig. S5. Enrichment of contaminant transcripts before MisTIC correction. Dot plots showing enrichment of contaminant transcripts among DEGs identified from uncorrected data, validated against LUAD scRNA-seq datasets. (a) Fibroblast vs. endothelial cell comparison. (b) Macrophage vs. T cell comparison. Dot size represents the percentage of cells expressing each gene; color intensity indicates average expression level.

Fig. S6 Cell-cell communication detection results on CoxMX data. (a) The CosMX pancreas SRT dataset with cell type annotation (b) Percentage changes in expression profiles by sender-receiver type with two negative control groups by contact-based CCC and secretion-based CCC.

Table S1 Datasets used in this study

File S1 Mathematical details of the MisTIC probabilistic model and additional analyses results

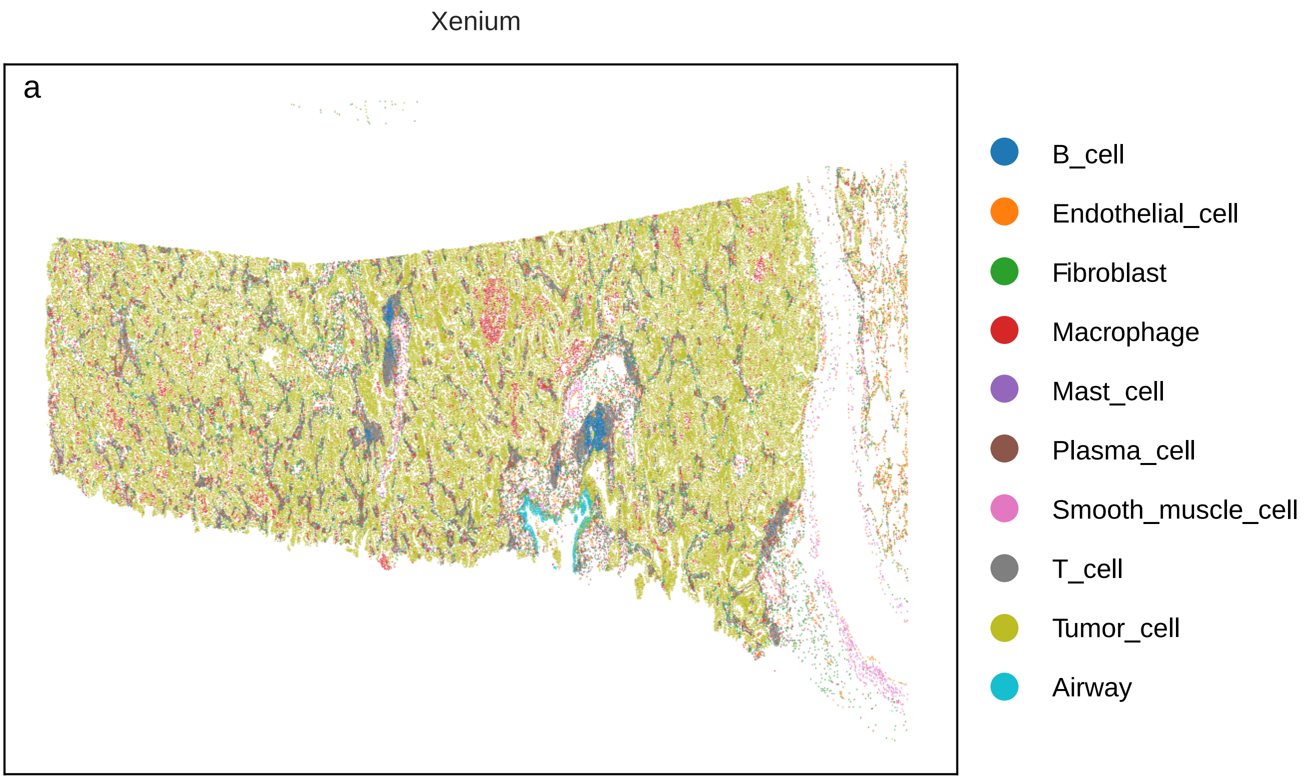
Xenium
a
B_cell
Endothelial_cell
Fibroblast
Macrophage
Mast_cell
Plasma_cell
Smooth_muscle_cell
T_cell
Tumor_cell
Airway

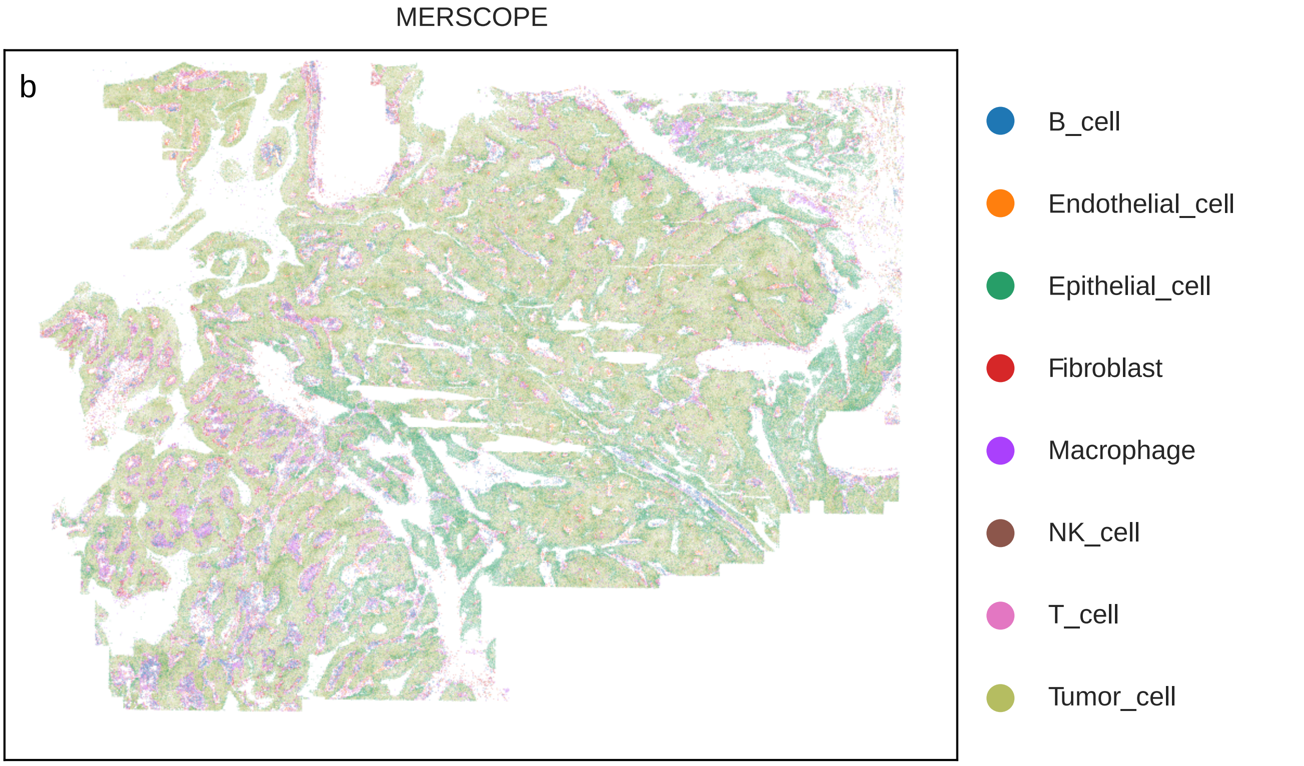
MERSCOPE
b
B_cell
Endothelial_cell
Epithelial_cell
Fibroblast
Macrophage
NK_cell
T_cell
Tumor_cell

bioRxiv preprint doi: https://doi.org/10.64898/2025.12.11.693759; this version posted December 15, 2025. The copyright holder for this preprint (which was not certified by peer review) is the author/funder, who has granted bioRxiv a license to display the preprint in perpetuity. It is made available under aCC-BY 4.0 International license.

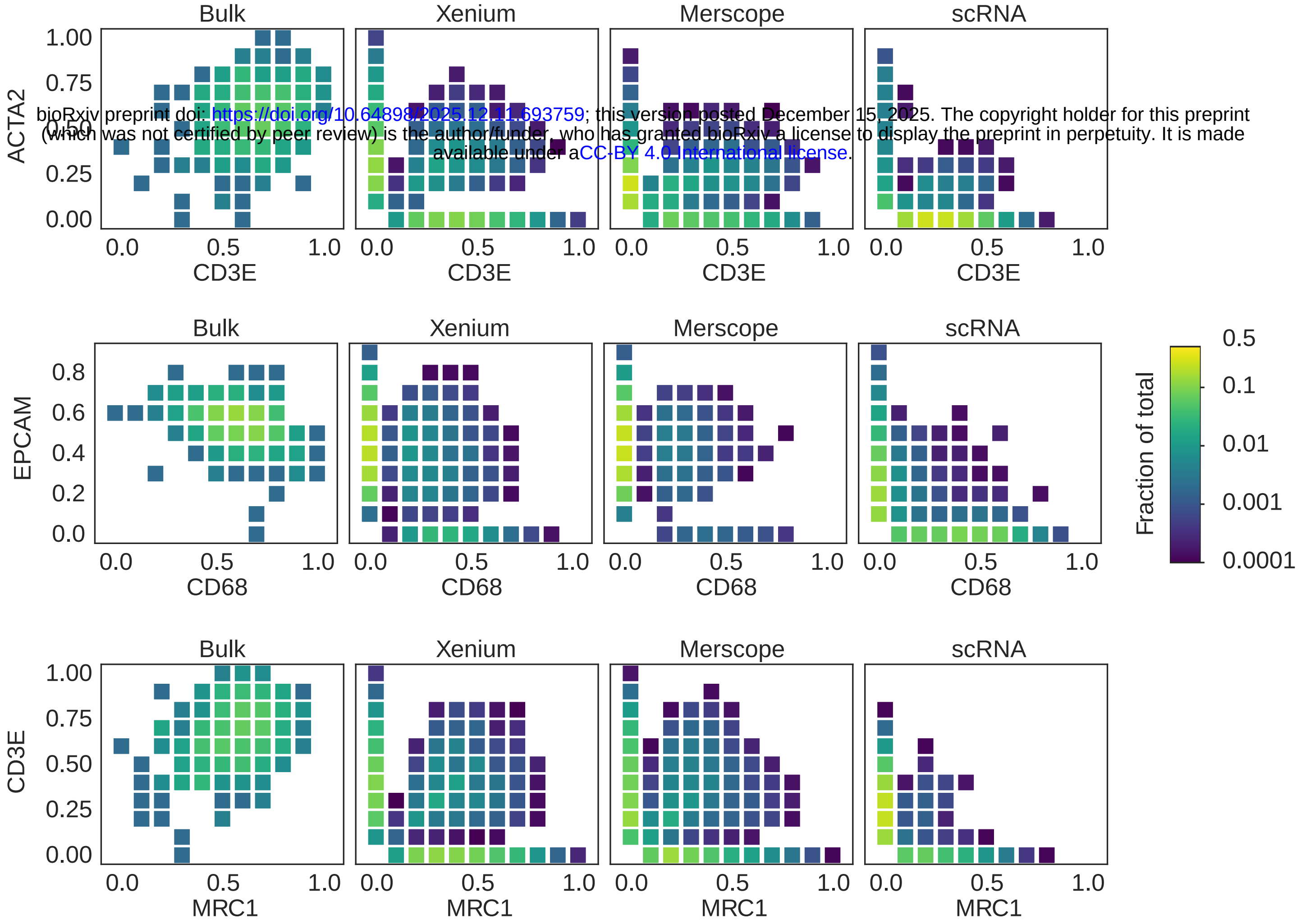

bioRxiv preprint doi: https://doi.org/10.64898/2025.12.11.693759; this version posted December 15, 2025. The copyright holder for this preprint (which was not certified by peer review) is the author/funder, who has granted bioRxiv a license to display the preprint in perpetuity. It is made available under aCC-BY 4.0 International license.

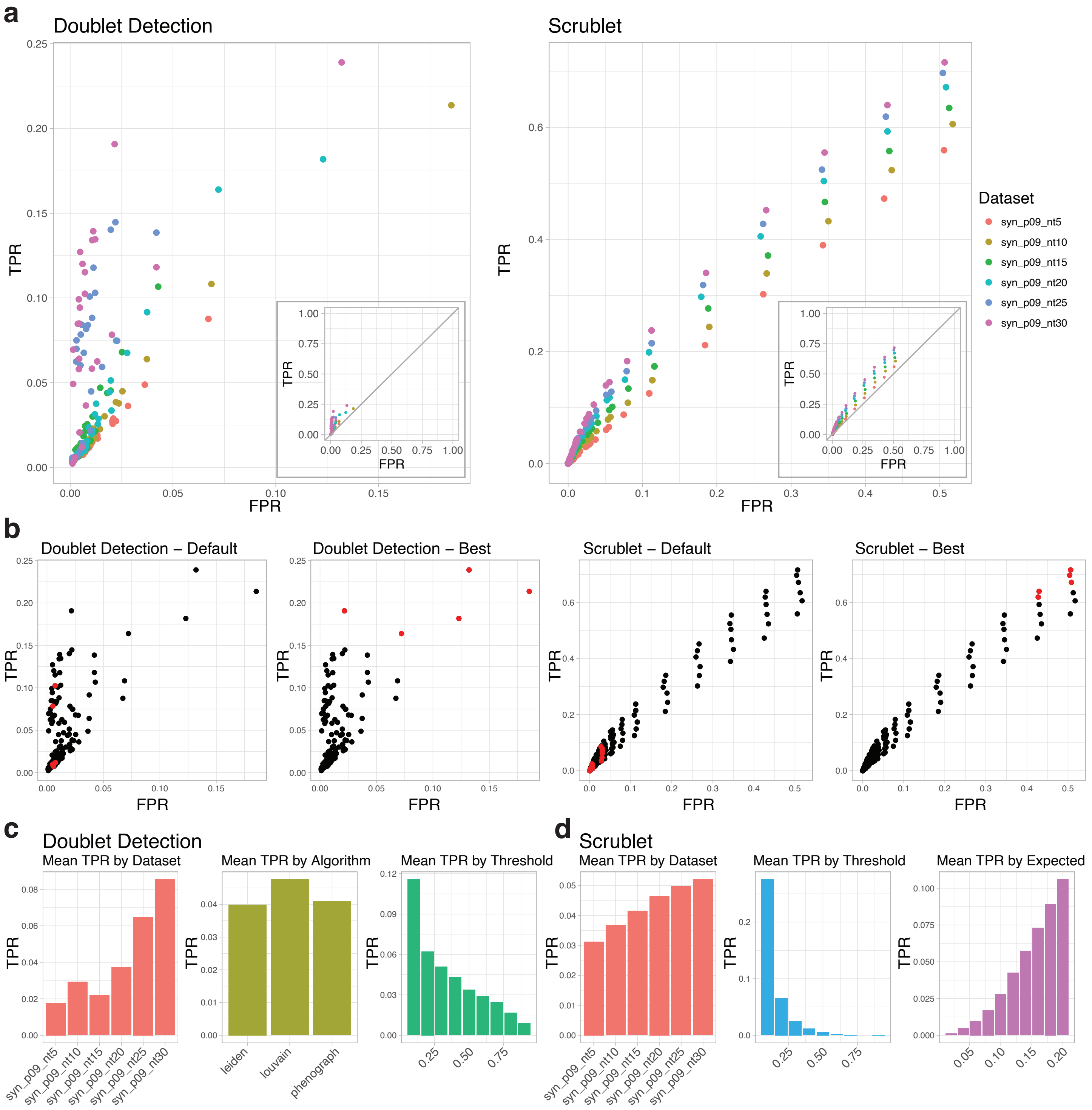

bioRxiv preprint doi: https://doi.org/10.64898/2025.12.11.693759; this version posted December 15, 2025. The copyright holder for this preprint (which was not certified by peer review) is the author/funder, who has granted bioRxiv a license to display the preprint in perpetuity. It is made available under aCC-BY 4.0 International license.

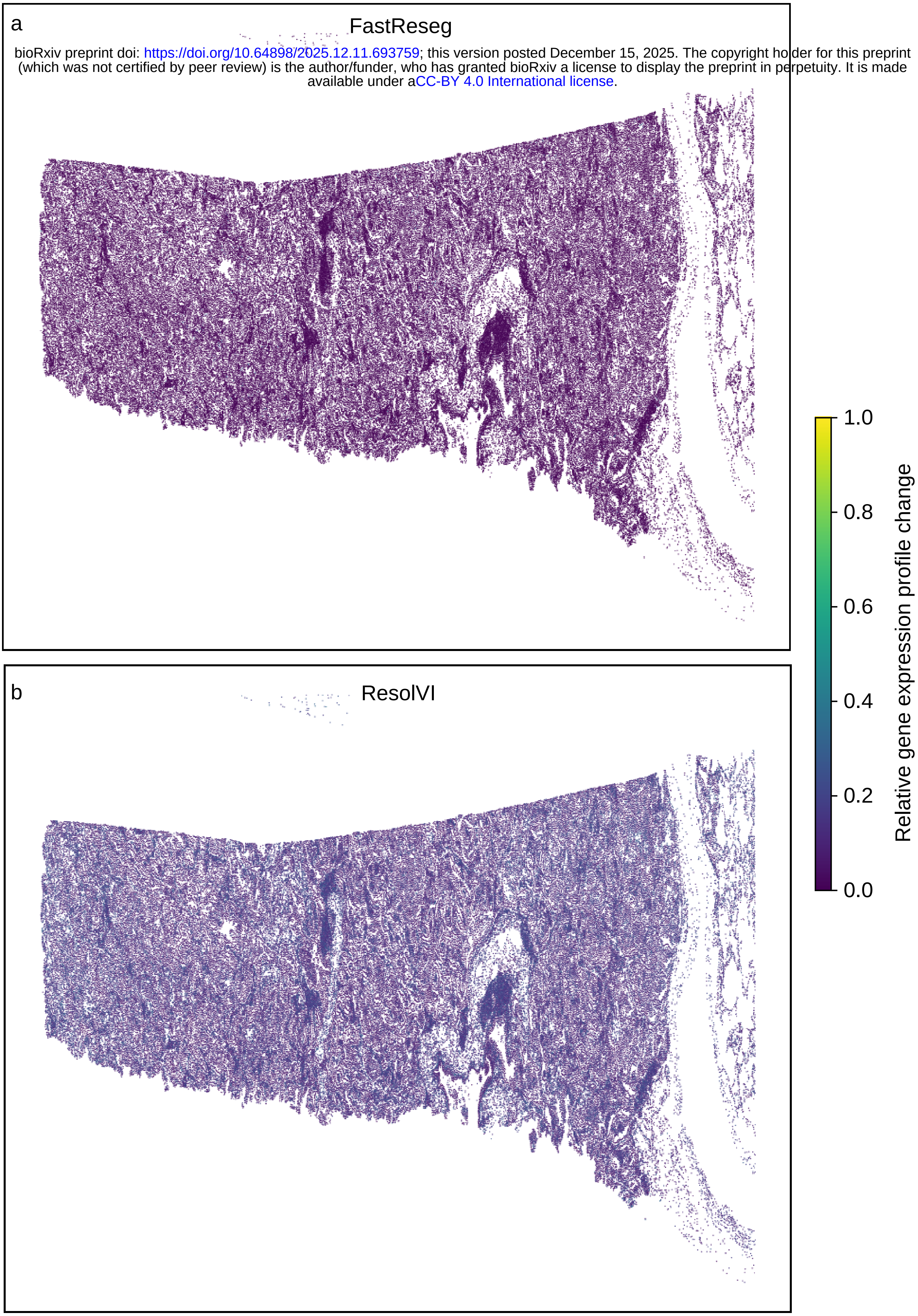

bioRxiv preprint doi: https://doi.org/10.64898/2025.12.11.693759; this version posted December 15, 2025. The copyright holder for this preprint (which was not certified by peer review) is the author/funder, who has granted bioRxiv a license to display the preprint in perpetuity. It is made available under aCC-BY 4.0 International license.

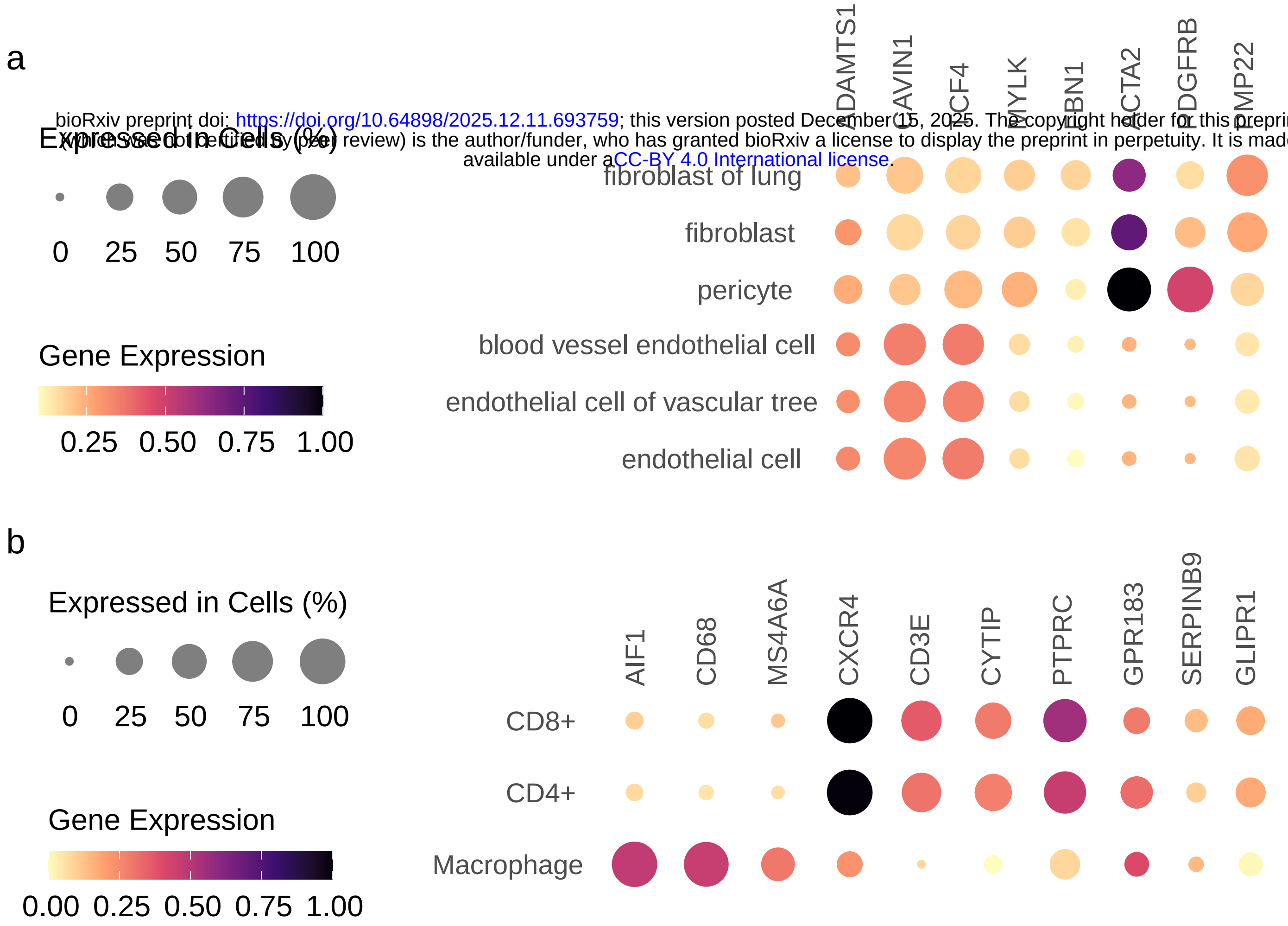

bioRxiv preprint doi: https://doi.org/10.64898/2025.12.11.693759; this version posted December 15, 2025. The copyright holder for this preprint (which was not certified by peer review) is the author/funder, who has granted bioRxiv a license to display the preprint in perpetuity. It is made available under aCC-BY 4.0 International license.

a

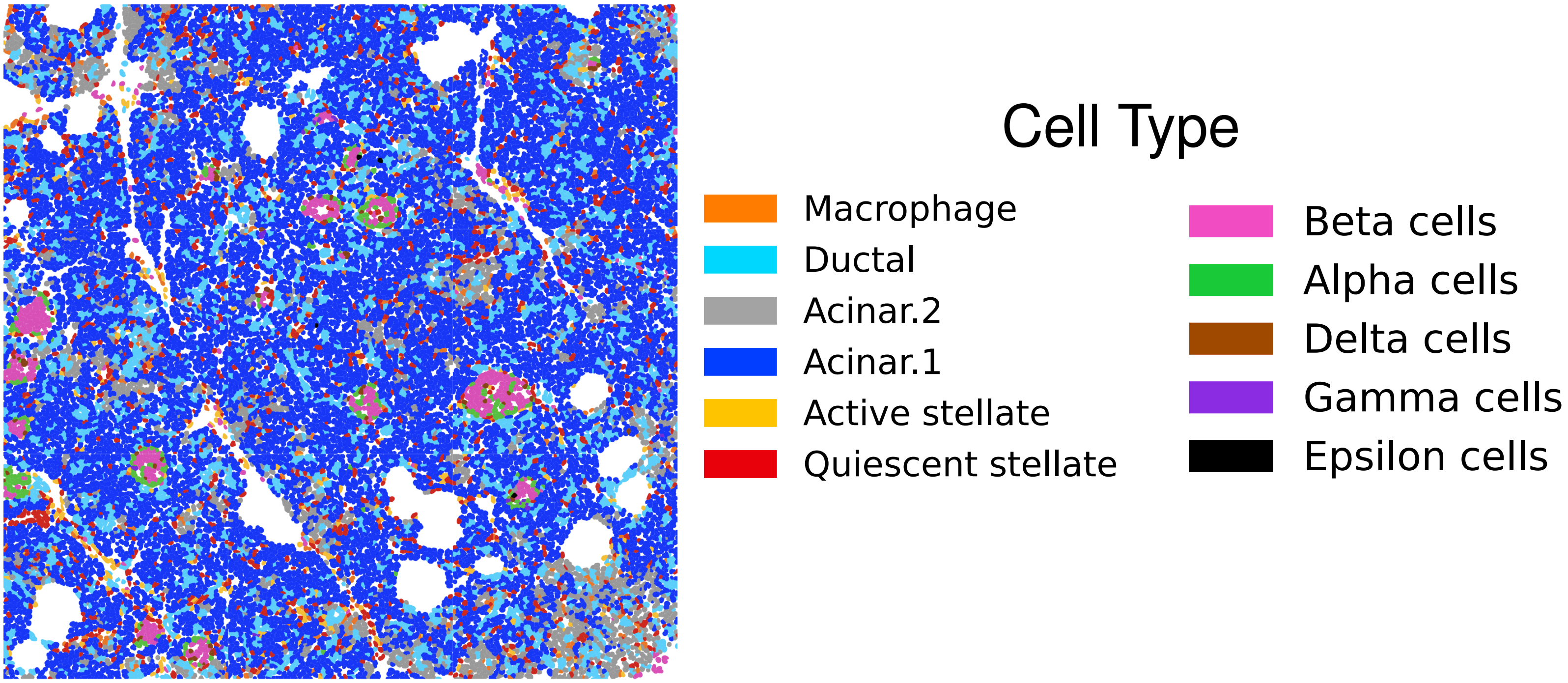

b

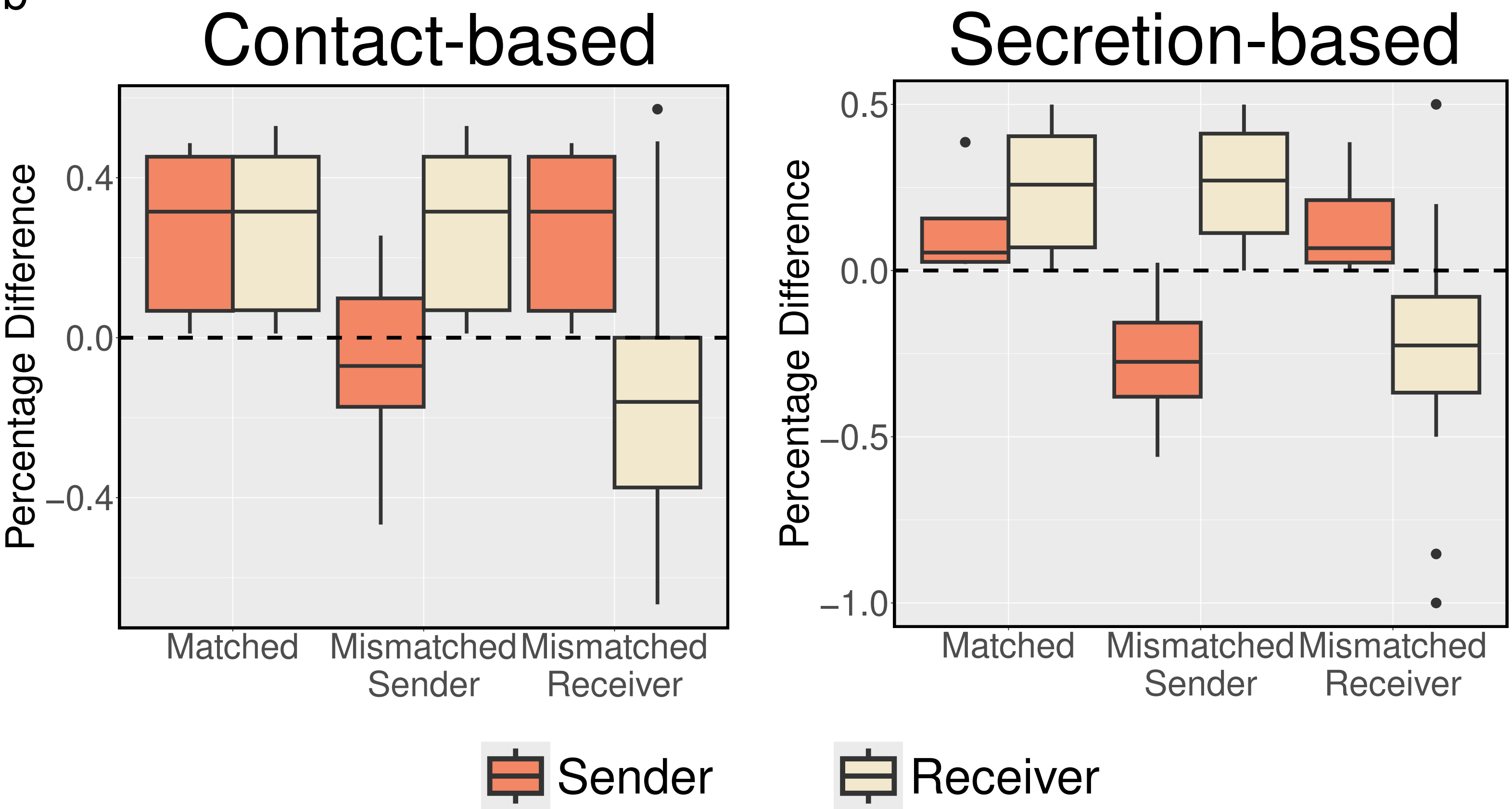

Supplement: Supplement 3 [file NIHPP2025.12.11.693759v1-supplement-3.pdf]
